# Don’t judge toxic weeds on whether they are native but on their ecological effects

**DOI:** 10.1002/ece3.6609

**Published:** 2020-07-29

**Authors:** Zhenchao Zhang, Jian Sun, Miao Liu, Ming Xu, Yi Wang, Gao‐lin Wu, Huakun Zhou, Chongchong Ye, Dorji Tsechoe, Tianxing Wei

**Affiliations:** ^1^ Synthesis Research Centre of Chinese Ecosystem Research Network Key Laboratory of Ecosystem Network Observation and Modelling Institute of Geographic Sciences and Natural Resources Research Chinese Academy of Sciences Beijing China; ^2^ State Key Laboratory of Soil Erosion and Dryland Farming on the Loess Plateau Institute of Soil and Water Conservation Northwest A&F University Yangling China; ^3^ Northwest Institute of Plateau Biology Qinghai Provincial Key Laboratory of Restoration Ecology of Cold Area Chinese Academy of Sciences Xining China; ^4^ Department of Ecology, Evolution, and Natural Resources School environmental and Biological Sciences Rutgers University New Brunswick NJ USA; ^5^ Institute of Tibetan Plateau Research Chinese Academy of Sciences Beijing China; ^6^ School of Soil and Water Conservation Beijing Forestry University Beijing China

**Keywords:** adaptive strategy, degraded grassland, ecological function, grassland management, toxic weed

## Abstract

The sharp rise in anthropogenic activities and climate change has caused the extensive degradation of grasslands worldwide, jeopardizing ecosystem function, and threatening human well‐being. Toxic weeds have been constantly spreading in recent decades; indeed, their occurrence is considered to provide an early sign of land degeneration. Policymakers and scientific researchers often focus on the negative effects of toxic weeds, such as how they inhibit forage growth, kill livestock, and cause economic losses. However, toxic weeds can have several potentially positive ecological impacts on grasslands, such as promoting soil and water conservation, improving nutrient cycling and biodiversity conservation, and protecting pastures from excessive damage by livestock. We reviewed the literature to detail the adaptive mechanisms underlying toxic weeds and to provide new insight into their roles in degraded grassland ecosystems. The findings highlight that the establishment of toxic weeds may provide a self‐protective strategy of degenerated pastures that do not require special interventions. Consequently, policymakers, managers, and other personnel responsible for managing grasslands need to take appropriate actions to assess the long‐term trade‐offs between the development of animal husbandry and the maintenance of ecological services provided by grasslands.

## Graphical abstract

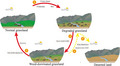

## 1 FOREWORD

Toxic weeds refer to plants of secondary compounds which are toxic to livestock, wild herbivores, and human (James et al., 2005). Some toxic weeds accumulate toxins at high levels whose concentration can be influence by the inhabiting conditions (Zhao, Gao, Wang, He, & Han, 2013). The toxic principles mainly include toxic proteins, terpenoids, glycosides, alkaloids, polyphenols, and photosensitive substances (Zhao et al., 2013), which can be extracted and used as pesticides with remarkable pesticidal and antimicrobial activities (Chen et al., 2017; Gao et al., 2013; Zhang, Jin, et al., 2011). As an indicator of grassland health, toxic weeds have become increasingly global in their distribution in recent decades indicating that the widespread land degradation is a serious issue that threatens the sustainable developmental goal of “no poverty, zero hunger” of the Food and Agriculture Organization of the United Nations (Sun et al., 2009; Wu, Han, Lu, & Zhao, 2016; Zhao et al., 2010, 2013). Furthermore, a longer growing season and warming induced by climate change will intensify the increases in the occurrence and production of toxic weeds (Klein, Harte, & Zhao, 2007; Su et al., 2019; Ziska, Epstein, & Schlesinger, 2009).

There are approximately 1,300 toxic species in over 140 families covering approximately 33.3 million hm^2^ in China's natural grasslands (Shi & Wang, 2004; Zhao et al., 2010). They have been traditionally thought that the wide distribution of toxic weeds leads to pasture degeneration and thereby reductions of grassland forage availability (Wu et al., 2016; Zhao et al., 2013). Additionally, poisonous weeds not only damage livestock breeding (Panter, James, Stegelmeier, Ralphs, & Pfister, 1999) but also poison—or even kill—domestic animals if they are ingested by accident or if the pollen is inadvertently inhaled (Bourke, 2007; Braun, Romero, Liddell, & Creamer, 2003; Zhao et al., 2013), potentially resulting in substantial economic losses and hindering the sustainable development of the livestock industry (Guo et al., 2017). About 300 of the 1,300 species of poisonous plants found in China exhibited negative effects on livestock (Shi, 1997). It is estimated that toxic weed poisoning results in direct or indirect economic losses of billions of CNY in China each year (Shi, 1997). The reduced grazing capacity and economic losses induced by toxic weed lead to lower resilience and increase in vulnerability of livelihoods that depend on livestock. Therefore, numerous approaches have been employed to control the spread of toxic weeds (Lu, Wang, Zhou, Zhao, & Zhao, 2012; Stokstad, 2013). However, most techniques have done little to eradicate established plants, and some approaches may even have negative environmental effects (Boutin, Strandberg, Carpenter, Mathiassen, & Thomas, 2014; Stokstad, 2013).

In fact, the spread of toxic weeds is not the reason for grassland degradation but a consequence of their strong adaptive capacity. Toxic weeds often have long and well‐developed root systems to facilitate the capture of water and nutrients from deep soil profiles (Sun, Wang, Cheng, Chen, & Fan, 2014), inhibit the growth of co‐occurring plants via allelopathy (Yan et al., 2016), form intraspecific aggregations that enhance their ability to compete with heterospecific competitors (Ren, Zhao, & An, 2015), and are not exposed to selection by livestock and small rodents (Zhao et al., 2013). From an ecological perspective, the colonization of toxic weeds might be more beneficial than harmful by promoting the process of succession in degraded grasslands by excluding excessive disturbance from livestock (Cheng, Sun, et al., 2014). An improved understanding of the potential role of toxic weeds in grassland conservation will challenge the traditional view that toxic weeds are uniformly deleterious and will enable pasture managers and policymakers to modify and design more flexible strategies for addressing global change and promoting sustainability. Here, we conduct a review of the literature to detail the fitness and potential effects of toxic weeds. These findings provide novel insight into the adaptive management of weed‐dominated grasslands.

## 2 ADAPTATIONS OF TOXIC WEEDS

In addition to the effects of natural factors, such as soil physiochemical properties and topographical conditions (Hou, Zhao, Li, Zhang, & Ma, 2013; Li et al., 2013), toxic weeds are most commonly a product of overgrazing and grassland degeneration. Previous studies have revealed that the population gradually increases and becomes dominant in plant communities as grassland degradation and grazing intensity increase (Li, Jia, & Dong, 2006; Ricciardi et al., 2017; Wang et al., 2016; Zhang, Yue, & Qin, 2004; Zhang, Yue, Qin, & Xue bin, 2004). This pattern is mostly due to that toxic weed has various strategies including higher genetic variation, well‐developed roots, allelopathy effect, and poisonous for herbivores adapting to environmental stress and anthropogenic disturbance.

### 2.1 Adaptive strategies to the environment

**FIGURE 1:**
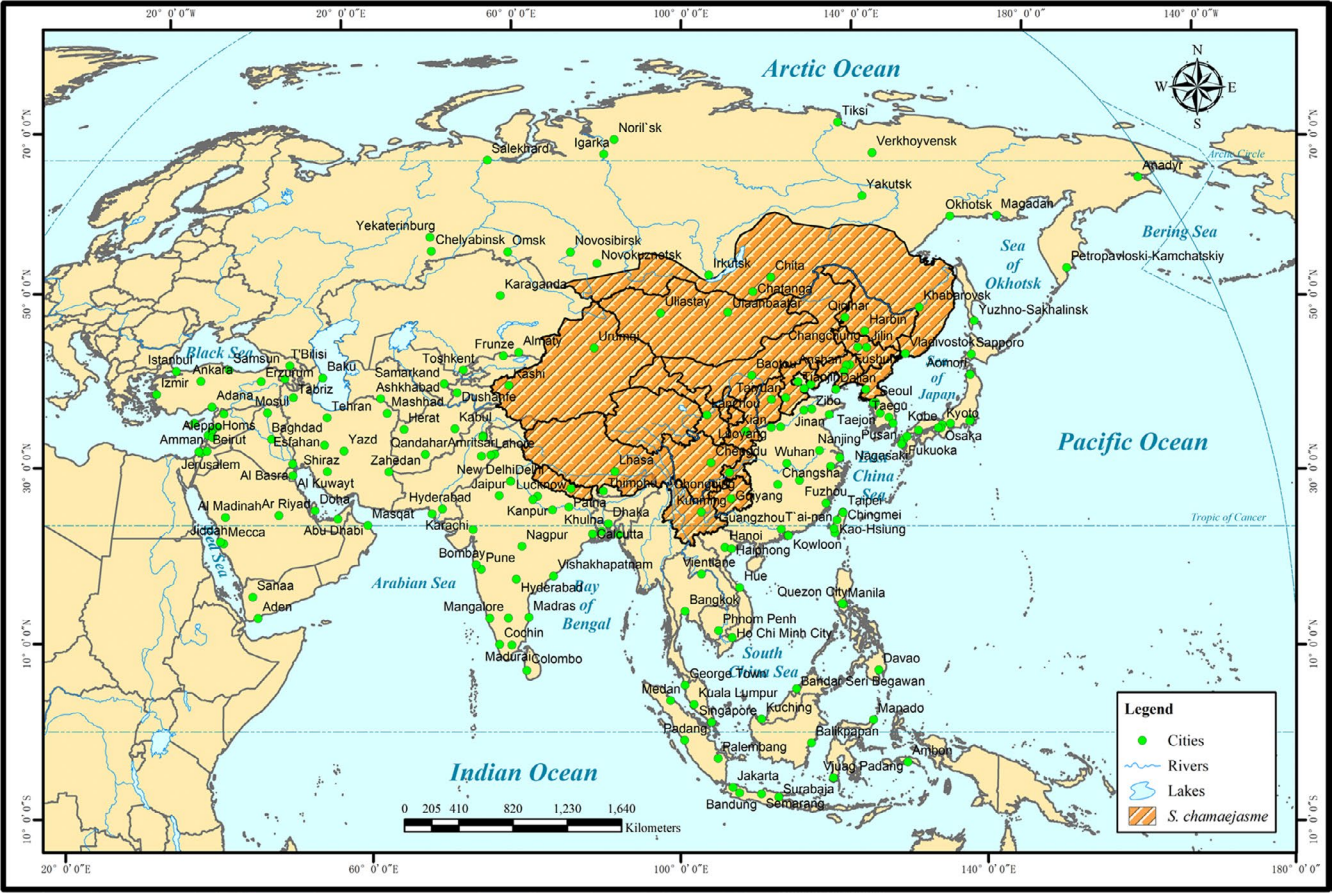
Global distribution of *S. chamaejasme* based on previously published records (Liu, Long, & Yao, 2004; Wang, 2004; Wang & Gilbert, 2007; Zhang, Volis, & Sun, 2010; Zhao et al., 2010), primarily including southern Russia, North Korea, Mongolia, Nepal, and northern and southwestern China

**FIGURE 2:**
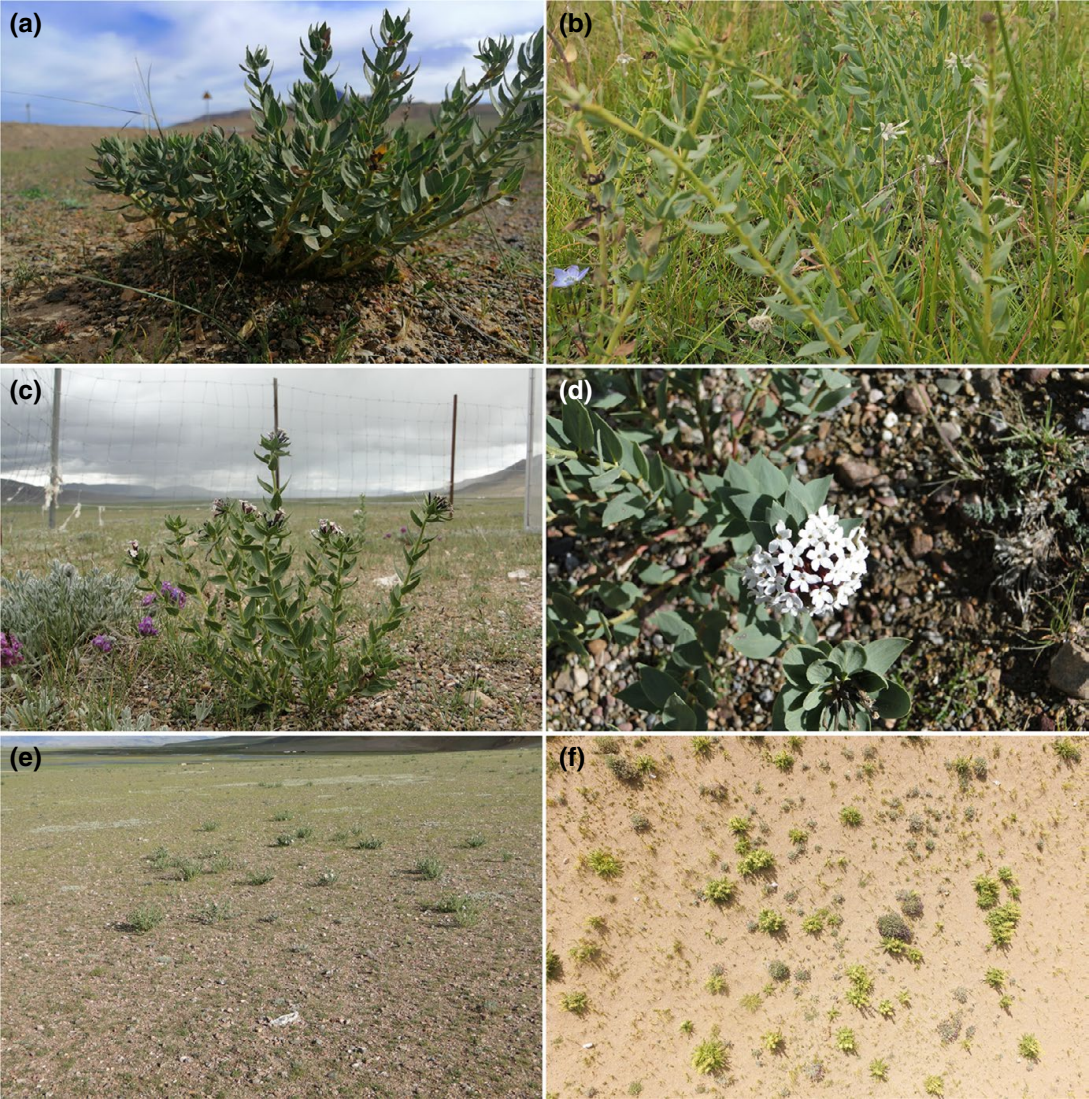
Plants, flowers, and landscapes of the toxic weed (*S. chamaejasme*). (a) plants of *S. chamaejasme* in an alpine grassland; (b) plants of *S. chamaejasme* in a typical grassland; (c) *S. chamaejasme* outside the fence; (d) white flower of *S. chamaejasme*; (e) landscape of *S. chamaejasme* in an alpine grassland; (f) landscape pattern of *S. chamaejasme* in a desert grassland taken by an unmanned aerial vehicle

A large number of toxic weeds are long‐lived perennial species with self‐incompatible mating systems and therefore generally have high genetic variation, which facilitates adaptive evolution to various environmental conditions and contributes to their wide geographic distribution (Bruijning, Metcalf, Jongejans, & Ayroles, 2020; Ghalambor, Mckay, Carroll, & Reznick, 2007; Zhang, Zhang, Li, & Sun, 2015). For example, *Stellera chamaejasme* inhabits a wide range of altitudes from 130 to 4,200 m, including a broad area from southern Russia to southwest China and the western Himalayas, which is suggestive of high adaptability (Figure 1). The various morphological and physiological traits of toxic weeds promote increases in the fitness to harsh environmental conditions, such as drought, cold, or barren soils (Kraft et al., 2015; Wang et al., 2016; Wong et al., 2004). As shown in Figure 2, leaves of these weeds are often lanceolate with thick waxy layers that tolerate prolonged drought conditions (Dou, Feng, & Hou, 2013). Moreover, many toxic weeds can capture water and nutrients from deeper soil profiles via their long and deeply distributed roots (Sun et al., 2009). Additionally, rhizobacteria has been found to stimulate the growth of these weeds by optimizing nutrient supplies and promoting plant metabolism and systemic resistance under unsuitable growth conditions (Cui et al., 2015; Hui et al., 2018; Lehmann et al., 2011; Lugtenberg & Kamilova, 2009). Endophytic bacteria also make some toxic weeds more tolerant to abiotic stress (Hyde & Soytong, 2008; Jin et al., 2014; Sieber, 2007).

Toxic weeds follow the optimal partitioning rule wherein plants partition photosynthate among their various organs to maximize growth rate in different habitats (Chapin, Bloom, Field, & Waring, 1987; Sun et al., 2019). For example, some toxic weeds have been observed to allocate more biomass to hydrotropic roots under drought stress (Sun et al., 2014). In addition, plant body size decreases at higher elevations to reduce nutritional needs in less resource‐rich environments; however, more photosynthetic products are allocated to flowers at higher elevations to enhance reproductive success (Zhang, Zhao, Ma, Hou, & Li, 2013). High altitudes make some toxic weeds produce fewer, but larger, flowers with color polymorphisms to attract pollinators in adverse environments (Zhang, Zhao, et al., 2015; Zhang et al., 2013) where low temperatures and strong winds discourage insect activity (Zhang, Zhang, & Sun, 2011). Also, the number of branches on toxic weeds is reduced and plant height is increased in north‐facing compared with south‐facing slopes, suggesting that toxic weeds allocate more photosynthate to vertical growth than to horizontal growth in response to competition for light (Hou, Zhao, Yu, Qian, & Ma, 2014). The physiological responses of toxic weeds also show signatures of adaptation to resource‐constrained conditions. For example, toxic weeds have higher rates of water use and proline concentrations which is conducive to a stronger resistance against adversity stress in south‐facing slopes with arid environments (Hou, Liu, & Sun, 2017; Liu & Ma, 2010). However, those in north‐facing slopes with weaker light intensities have higher chlorophyll contents and photosynthetic efficiencies (Liu, Zhao, Zhang, Li, & Shao, 2017).

### 2.2 Interspecific relationships

Owing to their wide niche breadth, toxic weeds can successfully coexist with several other plant species (Cheng, Chen, Yang, Xu, & Wang, 2014; Ren, Zhao, & An, 2013). Unlike the shallow‐rooted graminoids whose roots horizontally extend in the surface soil (Wang, Wang, Long, Jing, & Shi, 2004), toxic weeds are mostly axial‐root species which deeply root, and thus can absorb water and nutrients from much deeper in the soil compared to forages (Li, Niu, & Du, 2011; Maguire, Sforza, & Smith, 2011; Sun et al., 2014). Such interspecific differentiation in the acquisition of soil resources alleviates competition and permits co‐existence with heterospecific plants (Fargione & Tilman, 2006; Ryel, 2010; Xin et al., 2012). Nevertheless, perennial toxic weeds are usually tall and thus superior competitors for light resources relative to shorter plant species (Craine & Dybzinski, 2013; Hautier, Niklaus, & Hector, 2009; Li et al., 2016). In addition, individuals often aggregate to form patches that facilitate intraspecific cooperation, enhance their competitive ability, and promote their expansion (Gao & Zhao, 2013; Ren et al., 2015; Sun, Ren, & He, 2011). As a consequence, patches of heterospecific plants that are separated by toxic weeds often are not able to survive in the presence of competitively superior toxic weeds (Zhao, Gao, Wang, Sheng, & Shi, 2016).

**FIGURE 3:**
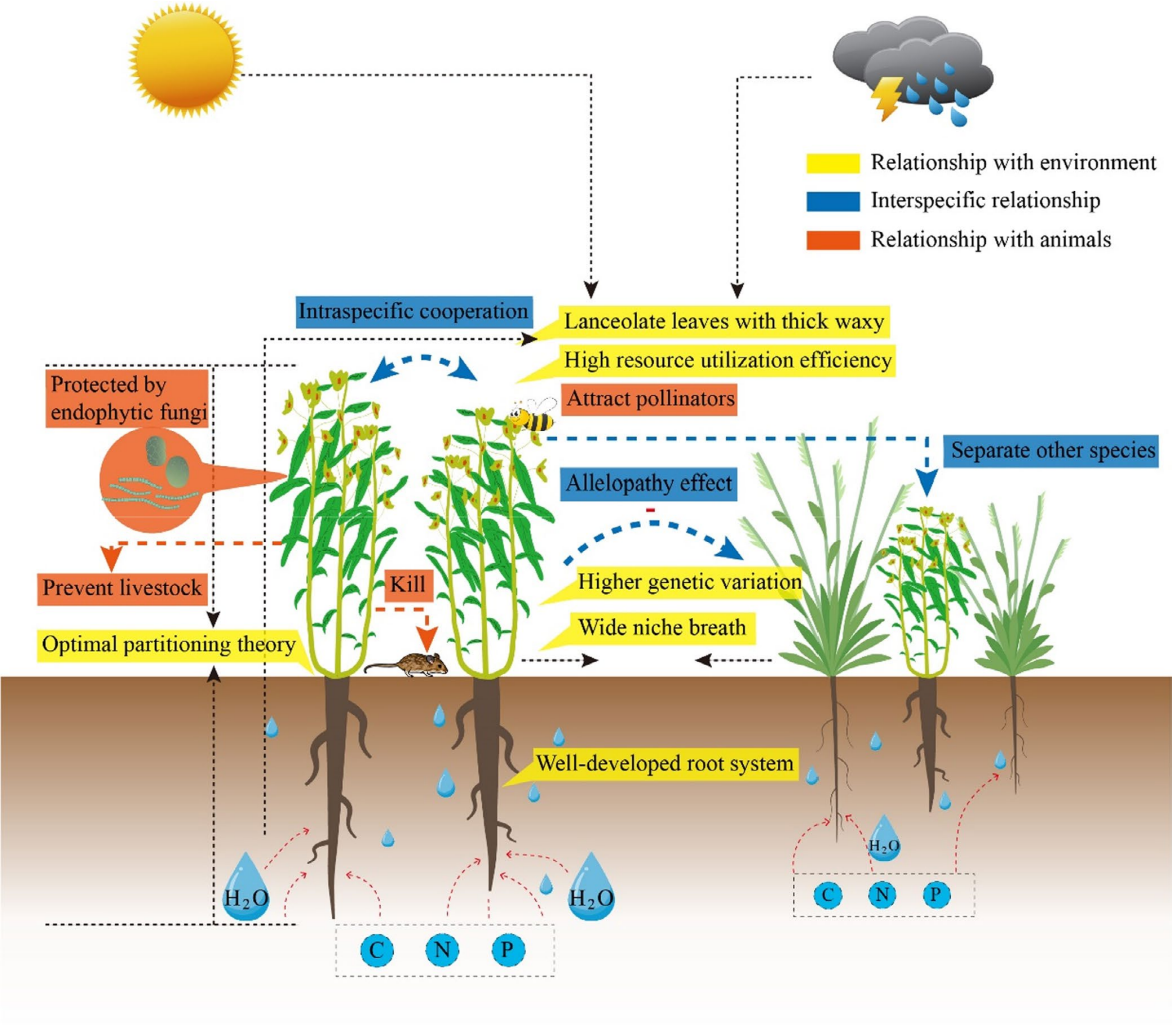
Conceptual graph of the adaptive strategies of toxic weeds for environmental stress (yellow background), competition from other plants (blue background), and animal disturbance (orange background). Fine dotted arrow = impacts of environmental conditions; thick blue dotted arrow = intraspecific and interspecific relationships; thick orange dotted arrow = interactions between plant and animals

The allelopathy is an important competitive behavior of some toxic weeds that inhibits the growth of their surrounding receptor plants (Figure 3). Most studies on allelopathy were done under laboratory conditions which is a serious caveat in allelopathy research. Here, we sorted the studies done under both laboratory and field conditions, and found that the primary phytotoxic mechanisms were regulated via the following two pathways. First, allelochemicals (e.g., flavonoids, coumarins, and phenolic compounds) can inhibit mitosis (Yan et al., 2016), reduce chlorophyll content (Pan, Li, Yan, Guo, & Qin, 2015), disrupt root development (Yan et al., 2014), promote the overproduction of proline (Yan et al., 2016), inhibit germination (Cheng et al., 2011), reduce endogenous auxin content (Yang et al., 2011), and promote reactive oxygen species accumulation (Pan et al., 2015; Yan, Zeng, Jin, & Qin, 2015).The second pathway is the arrest of sexual multiplication by pollen allelopathy (Sun, Luo, & Wu, 2010). Interestingly, phytotoxic effects increase with age; that is, older plants are superior competitors compared with younger plants (Wei, Zhong, Xu, Du, & Sun, 2017).

Notably, the allelopathy effects of toxic weeds exhibit species specificity; for example, *S. chamaejasme* has strong inhibitive effects on some species including *Setaria viridis*, *Amaranthus retroflexus* (Pan et al., 2015), *Pedicularis kansuensis* (Hou, Chen, Ren, Du, & Shang, 2011), *Festuca rubra* L., *Medicago sativa* (Guo et al., 2015), *Melilotus suaveolens Ledeb* (Wang, Zhou, & Huang, 2009), and *Onobrychis viciifolia* (Zhou, Huang, Wang, Liu, & Hui‐Fang, 2009), while other species such as *Agropyron mongolicum* (Wang, Zhou, Huang, Liu, & Hu, 2008), *Psathyrostachys juncea* (Zhou, Huang, Wang, Liu, & Hu, 2009), *Elymus dahuricus* (Zhou, Wang, Huang, & Liu, 2010), and *Lolium perenne* (Wang, Zhou, et al., 2009) show resistance against the allelopathy effect of *S. chamaejasme*. Therefore, these species can be used to restore degraded grasslands inhabited by toxic weeds.

### 2.3 Weed‐animal interactions

Toxic weeds are more resistant to grazing than grasses favored by herbivores, especially when available forage is limited (Ren, Li, Ouyang, Ma, & Dai, 2016). They also exhibit superior tolerance to physical breakdown because of their tenacious capacity to regenerate once damaged (Li et al., 2008). Endophytic fungi can protect plants from nematodes, insect pests, and fungal pathogens (Barillas, Paschke, Ralphs, & Child, 2007; Jin et al., 2013). Furthermore, the toxic compounds of these weeds are capable of poisoning or killing small rodents and play a vital role in protecting toxic weeds from animals and pathogens (Yan et al., 2015). The content of toxic substances is highest in leaves, which is the vegetative organ most likely to be consumed by herbivores. Furthermore, the content of toxic substances dramatically increases in response to trampling and consumption by livestock, which reduces the grazing intensity on toxic weeds (Zheng & Hu, 2006). The texture and color of toxic weeds are also striking (Figure 2), which likely aid the identification, recognition, and classification of toxic weeds by animals as distasteful and indigestible food items.

In response to long‐term overgrazing and selective foraging, palatable grasses would exhibit a dwarfing tendency, restricting their ability to utilize natural resources (Evju, Austrheim, Halvorsen, & Mysterud, 2009). However, the number of reproductive branches and individual florets of toxic weeds increase to ensure reproductive success under grazing condition (Han, Chen, & G. Y., Sun, J. & li, J. P., 2006). The grazing‐induced reduction of interspecific competition also contributes to the dominance of toxic weeds in plant communities (Ren et al., 2016). In addition to grazing duration, grazing intensity also affects the distribution of toxic weeds, which often aggregate when grazing is intense but are randomly distributed when grazing is especially intense (Xing & Song, 2002; Zhao, Gao, Sheng, Dong, & Zhou, 2011). Thus, the intraspecific relationship shifts from being mutualistic to competitive depending on the intensity of grazing (Ren & Zhao, 2013).

Reproductive strategies of toxic weeds with high survival rates include floral traits, such as the brilliant terminal flower head (Figure 2d). For instance, the flower colors of *Iris lacteal*, *Gentiana sino‐ornata*, *Consolida ajacis*, *Anaphalis sinica* are in sequence lavender, purple, blue, and while, which increase reproductive success by attracting pollinators (James et al., 2005; Zhang, Zhang, et al., 2011). Additionally, the seeds are hard and long‐lived and the seedlings are capable of exploiting grazed areas with reduced competition from palatable grasses (Zhao et al., 2013). The proportion of old plants in grasslands increases with grazing intensity. In addition, old individuals have a higher fecundity and produce larger quantities of seeds compared with younger plants (Xing, Gou, & Wei, 2004). Thus, the breadth and density of the soil seed bank increases as the intensity of grassland degradation rises, enhancing the ability of the population to regenerate (Du, Zhao, Song, & Shi, 2015; Zhao & Zhang, 2010).

## 3 POTENTIAL ECOLOGICAL EFFECTS OF TOXIC WEEDS

Traditionally, toxic weeds are not only thought to cause economic losses to livestock production but are also thought to do great harm to grasslands and lead to their degradation (James et al., 2005; Lu et al., 2012; Zhao et al., 2013). However, this parochial view may neglect the manifold ecological roles that toxic weeds can play as important natural components of grassland ecosystems. For instance, toxic weeds can provide a number of ecological, social, and economic benefits by improving soil quality, protecting forage resources, and promoting the sustainable development of grasslands.

### 3.1 Effects on soils

Regarding soil and water conservation, the well‐developed root systems of toxic weeds can fix sand and capture nutrients from soils with coarser textures (Wang, 2001; Wong et al., 2004). Grazing and grassland degradation induce reversed vegetation succession with deterioration of plant community structure from palatable grasses to toxic weeds (Wang, Long, Wang, Jing, & Shi, 2009; Wu, Du, Liu, & Thirgood, 2009). Even so, compared to bare land, grassland covered by toxic weeds is more susceptible to erosion from strong wind and rain (Zhang et al., 2004). On the other hand, toxic weeds significantly increase the water content of the soil surface under drought conditions (An et al., 2016). The higher coverage of plants shields topsoil from solar radiation and decreases evaporation (Mchunu & Chaplot, 2012); moreover, the soil infiltration rate is relatively high as a result of a well‐developed root system, stimulating rainfall storage (Song, Dong, Liu, & Liu, 2018).

**FIGURE 4:**
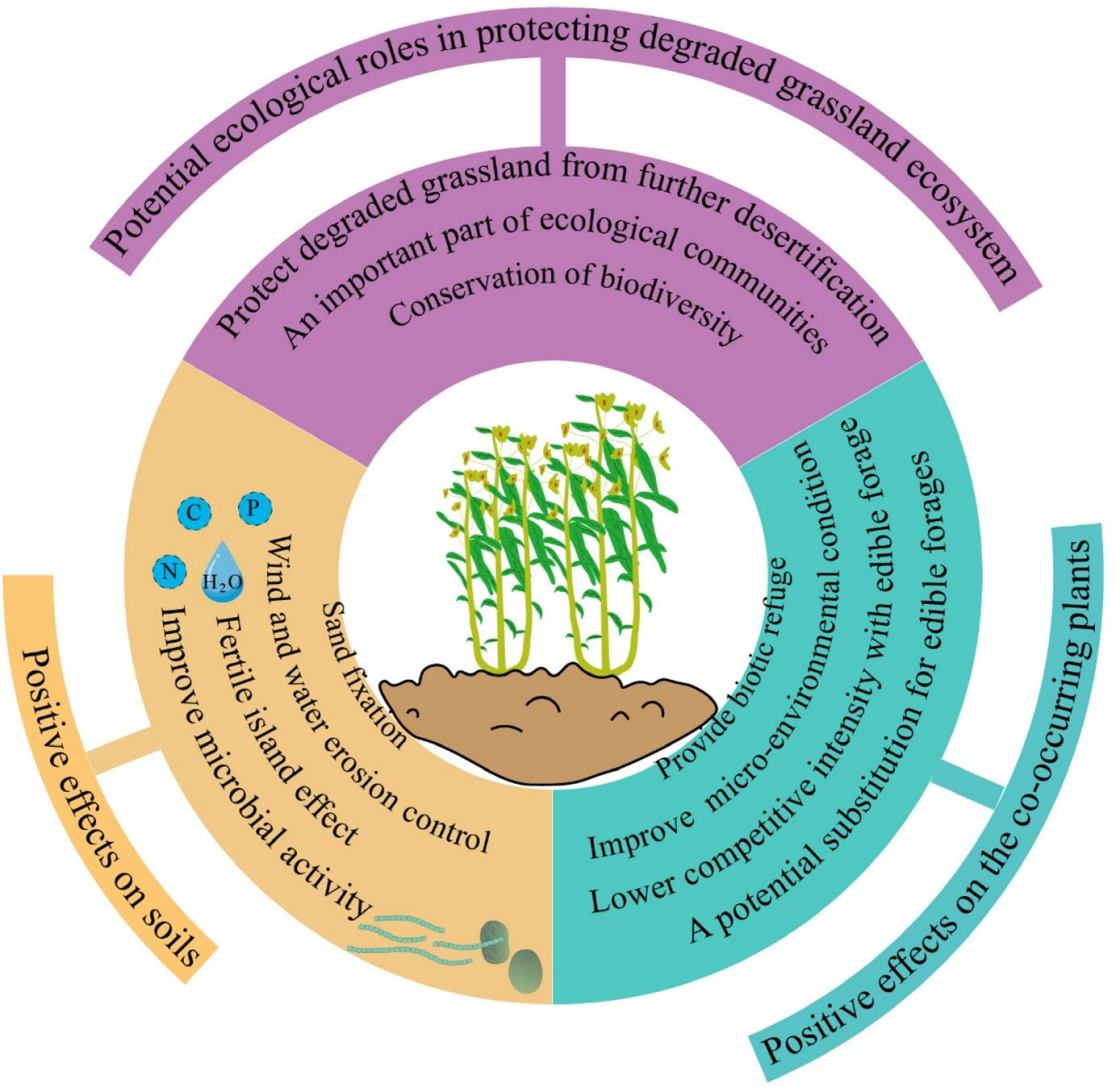
The potential ecological effects of toxic weeds on grassland ecosystems (purple background), soil (yellow background), and co‐existing plants (green background)

In addition to the physical protection that they provide to grasslands, toxic weeds have remarkable effects on soil nutrient pools and can create fertile islands (Sun et al., 2009) (Figure 4). Toxic weeds produce more litter as a consequence of their increased growth and because they lose less tissue through grazing. Toxic weeds are also more labile and have higher tissue nitrogen and lower lignin nitrogen compared with other species (An et al., 2016). Soil microorganisms also contribute to the turnover rate and nutrient availability. Soil microbial biomass and soil enzyme activities are higher in toxic weed patches than in areas between these patches (An et al., 2016). Overall, the protection and improvement of soil by toxic weeds provide a superior material basis for plant growth and benefit the recovery of degraded grasslands.

### 3.2 Effects on co‐occurring plants

It is commonly assumed that toxic weeds have negative effects on the quantity of forage via allelopathy, thereby decreasing grassland productivity (Pan et al., 2015). However, toxic weeds actually provide biotic refuges and keep surrounding herbaceous species away from livestock in overgrazed grasslands (Figure 4). Cheng, Sun, et al. (2014) found that the number of species and the coverage of neighboring plants are noticeably higher in plots with toxic weeds than in those in open grasslands. There are two principal means by which toxic weeds can facilitate the proliferation of neighboring plants in overgrazed pastures. First, the toxic smell could repel livestock and thus reduce the ingestion and trampling of edible forage surrounded by toxic weeds (Oesterheld & Oyarzabal, 2004). Second, toxic weeds alter the surrounding micro‐environmental conditions. For example, toxic weeds can redistribute soil nutrients, form fertility islands (Sun et al., 2009), and create a cool environment that promotes soil moisture retention via the height of the plant canopy (Rebollo, Milchunas, & Chapman, 2002). All of these micro‐environmental changes provide better soil conditions and microclimates for plant growth. Additionally, the niche overlap between toxic weeds and fine herbage is smaller than that between toxic weeds and unpalatable weeds, reflecting the lower degree of competition between toxic weeds and edible forage (Ren et al., 2013).

### 3.3 Potential ecological roles in degraded grasslands

**FIGURE 5:**
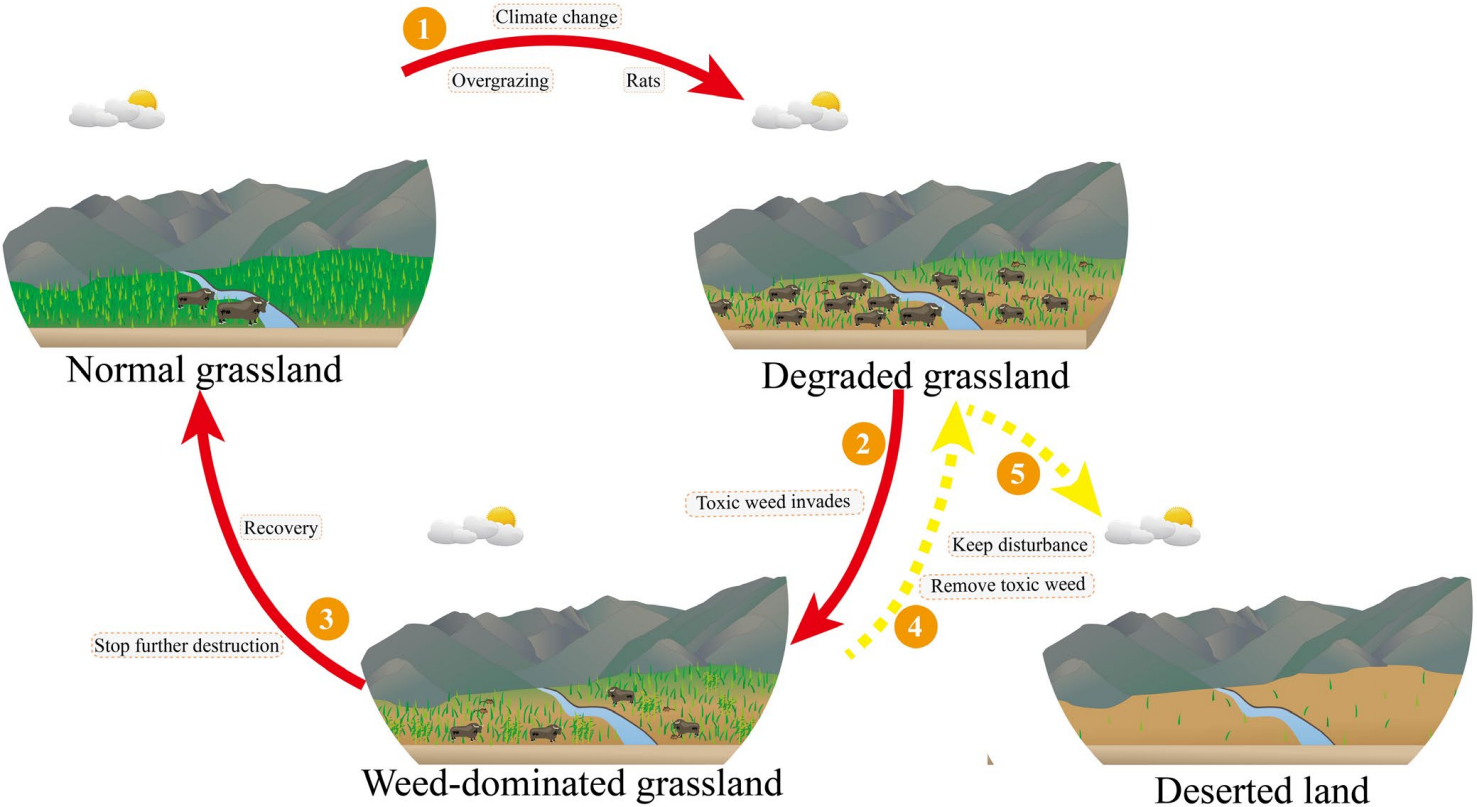
The processes of grassland succession. ① Grassland degrades as a result of climate change and human activities; ② Toxic weeds invade as a consequence of their many adaptations to disturbed environments; ③ Degraded grassland recovers under the protection of toxic weeds from excessive destruction; ④ Livestock and rats destroy degraded grasslands by the excessive removal of toxic weeds; ⑤ The grassland ecosystem collapses and desertification occurs as a consequence of the excessive damage. Red solid arrows indicate the positive feedback loop with toxic weeds. Yellow dotted arrows indicate the negative feedback direction that occurs in the absence of toxic weeds

From a successional perspective, the spread of toxic weeds is a consequence of their high adaptability rather than a cause of grassland degeneration. As an important part of the grassland ecosystem, toxic weeds improve plant community structure in degraded pastures (Tan & Zhou, 1995) and play a crucial role in preventing further desertification of degraded grasslands (Wang et al., 2016). Animals usually avoid poisonous toxic weeds, which inherently suppresses excessive disturbance by livestock when overgrazing occurs. The unfounded removal of toxic weeds might lead to ecosystem collapse (Figure 5) because grazing pressure on pasture is greater without the protection that toxic grasses provide (Holechek, 2002; Wang, Wang, Cheng, & Hou, 2014). This hypothesis is potentially consistent with previous studies that report that the degree of degradation of mowed grasslands was greater than that of grazed grasslands inhabited by toxic weeds (Li et al., 2008; Wang & Gilbert, 2007).

Furthermore, the presence of toxic weeds provides an essential means by which the coverage of vegetation can be maintained and the ecological functions of degraded grassland can be preserved (Figure 5), although these should be considered some of their “better‐than‐nothing” effects. Toxic weeds provide an important gene pool, and their invasion increases the diversity of insects and invertebrates, facilitating the maintenance of biodiversity (Sun, Chen, Zhao, & Long, 2013). Consequently, degraded grassland with toxic weeds does not require any special interventions aside from controlling grazing intensity or limiting the overgrowth of toxic weeds (Holechek, 2002). In support of these effects, the occurrence of toxic weeds is inhibited by the absence of grazing (Ren et al., 2016). The potential process and underlying mechanism are as follows: First, residual yak dung deposition accelerates the proportional increase in graminoids and promotes the transformation of grasslands to gramineous communities following the exclusion of grazing (Mou et al., 2013). Moreover, grasses will recolonize and regain prevalence due to the maintenance of local genetic variation and because they can regenerate rapidly through the production of a large number of seeds (Cheng, Sun, et al., 2014; Liu & Ma, 2010). We hypothesize that degraded grassland ecosystems will eventually be restored and become prosperous again following a long period of self‐healing (Figure 5).

## 4 CONCLUSIONS AND FUTURE PROSPECTS

An improved understanding of toxic weeds is valuable for the sustainable management of grasslands and for meeting the 2030 Global Land Degradation Neutrality Target set by the United Nations Convention to Combat Desertification (Toth, Hermann, Silva, & Montanarella, 2018). This review provides an understanding of the adaptive abilities of toxic weeds and presents a new interpretation of their role in degenerated grassland ecosystems. Here, we argue that toxic weeds can provide self‐protective mechanisms of degraded pastures and promote their resilience. In some cases, taking no action might be cost‐effective to taking actions that end up doing more harm than good. The blind removal of toxic weeds through the promotion of increased grazing will likely expose pastures to excessive damage, jeopardizing ecosystem balance. Thus, robust grassland management requires policymakers, managers and other personnel to continuously monitor and evaluate the long‐term trade‐offs between the development of livestock farming and the maintenance of multiple ecological services.

The limitation of this paper is that we focused on the potential positive effects of toxic weeds which have been largely neglected by conventional wisdom. Notably, an objective justification to treat these poisonous species differently must be based on the trade‐off of their positive and negative effects considering many aspects. However, due to the limited availability of studies, we were unable to make a quantitative assessment of the negative and positive effects of toxic weeds. Subsequent studies should allocate more efforts to quantify and assess the trade‐off between positive and negative effects of poisonous species, so as to adopt adaptive grassland management dealing with the presence of toxic weeds.

## CONFLICT OF INTEREST

The authors declare no conflict of interest.

## AUTHOR CONTRIBUTION

**Zhenchao Zhang:** Conceptualization (lead); Funding acquisition (supporting); Methodology (lead); Software (lead); Validation (lead); Writing‐original draft (lead); Writing‐review & editing (equal). **Jian Sun:** Conceptualization (lead); Funding acquisition (lead); Methodology (equal); Project administration (equal); Writing‐original draft (supporting); Writing‐review & editing (supporting). **Miao Liu:** Conceptualization (equal); Methodology (equal); Software (equal); Supervision (equal); Writing‐review & editing (equal). **Ming Xu:** Conceptualization (lead); Methodology (equal); Writing‐review & editing (supporting). **Yi Wang:** Resources (equal); Software (equal); Supervision (equal); Writing‐review & editing (equal). **Gaolin Wu:** Methodology (equal); Supervision (equal); Writing‐review & editing (equal). **H Zhou:** Formal analysis (equal); Supervision (equal); Writing‐review & editing (equal). **Chongchong Ye:** Investigation (equal); Supervision (equal); Writing‐review & editing (equal). **Tsechoe Dorji:** Supervision (equal); Writing‐review & editing (equal). **Tianxing Wei:** Resources (equal); Writing‐review & editing (equal).

## Data Availability

All data included in this study are available upon request by contact with the corresponding author.
